# A community-based study on the association between *Helicobacter pylori* Infection and obesity

**DOI:** 10.1038/s41598-018-28792-1

**Published:** 2018-07-16

**Authors:** Li-Wei Chen, Sheng-Fong Kuo, Chih-Hung Chen, Cheng-Hung Chien, Chih-Lang Lin, Rong-Nan Chien

**Affiliations:** 1 Department of Gastroenterology and Hepatology, Chang-Gung Memorial Hospital and University at Keelung, Keelung, Taiwan; 2 Community Medicine Research Center, Chang-Gung Memorial Hospital and University at Keelung, Keelung, Taiwan; 3 Department of Metabolism and Endocrinology, Chang-Gung Memorial Hospital and University at Keelung, Keelung, Taiwan

## Abstract

*Helicobacter pylori* (*H*. *pylori)* infection can induce chronic inflammation and is associated with insulin resistance, metabolic syndrome and body mass index (BMI, kg/m^2^) changes. This study aimed to evaluate the association between *H*. *pylori* infection and overweight/obesity. This research was a cross-sectional study conducted from March 2014 to November 2016, using data from the three districts in the northeastern region of Taiwan. The inclusion criteria were an age >30 years and the absence of pregnancy. Ultimately, 2686 subjects (1713 women) were included in this study. Among the subjects aged less than 50 years, the subjects with *H*. *pylori* infection had higher mean BMI values than those without *H*. *pylori* infection (40–49 years: 25.7 ± 4.4 vs. 24.7 ± 3.8, *P* = 0.025; 30–39 years: 24.9 ± 4.4 vs. 24.0 ± 4.1, *P* = 0.063). *H*. *pylori* infection increased the risk of being obese 2 (BMI ≥30) (odds ratio, OR = 1.836, 95% CI = 1.079–3.125, *P* = 0.025) with adjustments for demographic factors in subjects aged less than 50 years. In conclusions, subjects with *H*. *pylori* infection and age less than 50 years may increase a risk of being obesity (BMI ≥30) compared to those without this type of infection.

## Introduction

Overweight or obesity is a worldwide epidemic disease and a public health problem in Taiwan^1–4^. The prevalence of overweight in adults is 38.5% (men 48.7%, women 28.3%) based on the Taiwan criteria (body mass index, BMI ≥ 24 kg/m^2^)^3,4^. Obesity-related comorbidities, such as diabetes mellitus and hypertension, are a public health economic burden in Taiwan^5^. Excessive caloric intake and decreased physical activity are the main reasons for the increasing prevalence of obesity^6^. Recently, gut microbiota has been reported to have an important role in the development of obesity^7,8^. *Helicobacter pylori* (*H*. *pylori*) is a gram-negative microorganism found in the human stomach. Chronic infection with *H*. *pylori* will induce an immune response and result in local gastritis or a systemic response^9,10^. In recent studies, *H*. *pylori* was also found to be associated with some extradigestive diseases, such as insulin resistance, metabolic syndrome and obesity^11–13^. The mechanism associating *H*. *pylori* infection and obesity may be related to *H*. *pylori* infection-related gastritis or peptic ulcer, immunological cytokines and leptin^14–18^. In clinical observations, *H*. *pylori* infection-related gastritis or peptic ulcers have been found to lead to dyspepsia and poor appetite. Patients gain weight following successful *H*. *pylori* eradication^19–21^. Tumor necrosis factor-α (TNF-α) is a key mediator of inflammation that is involved in the development of obesity-related insulin resistance^22^. Leptin is an adipokine and might regulate body weight via decreased appetite and food intake^23^. Gastric inflammation is highest with cytotoxin-associated gene A (cagA) strains of *H*. *pylori*^24,25^. The prevalence of *H*. *pylori* infection is approximately 50–60% in people aged 50 years old, and most (99%) of the *H*. *pylori* infection strains are cagA-positive strains in Taiwan^26^. *H*. *pylori* infection can induce changes in gastric mucosal leptin and TNF-α levels, which influence body weight changes^10,14,16,27,28^. Recent cross sectional studies have reported conflicting results that demonstrate an association between *H*. *pylori* infection and BMI^13,29–34^. Most of these association studies lacked adjustments for confounding factors, such as socioeconomic class, education status, jobs, and mental or psychological evaluations, which are associated with BMI. It is necessary accurately adjust for these potential confounders to evaluate the association between *H*. *pylori* infection and BMI. We hypothesized that colonization with *H*. *pylori* is associated with a change in BMI due to chronic inflammation and insulin resistance and that cytokines (TNF-α and C reactive protein) and adipokines (adiponectin and leptin) are involved. Most patients get *H*. *pylori* infections during childhood, the inflammatory influences of *H*. *pylori* infection on body weight may be different between patients in the young age (early life) and in the old age (late life)^35^. This study aimed to evaluate the association between *H*. *pylori* infection and BMI using data from three districts in the northeast region of Taiwan. Data including age, inflammatory cytokine and adipokine levels as well as detailed demographic data (socioeconomic status, education, job, mental or psychological evaluation scores) were included in the analysis.

## Results

**Figure 1:**
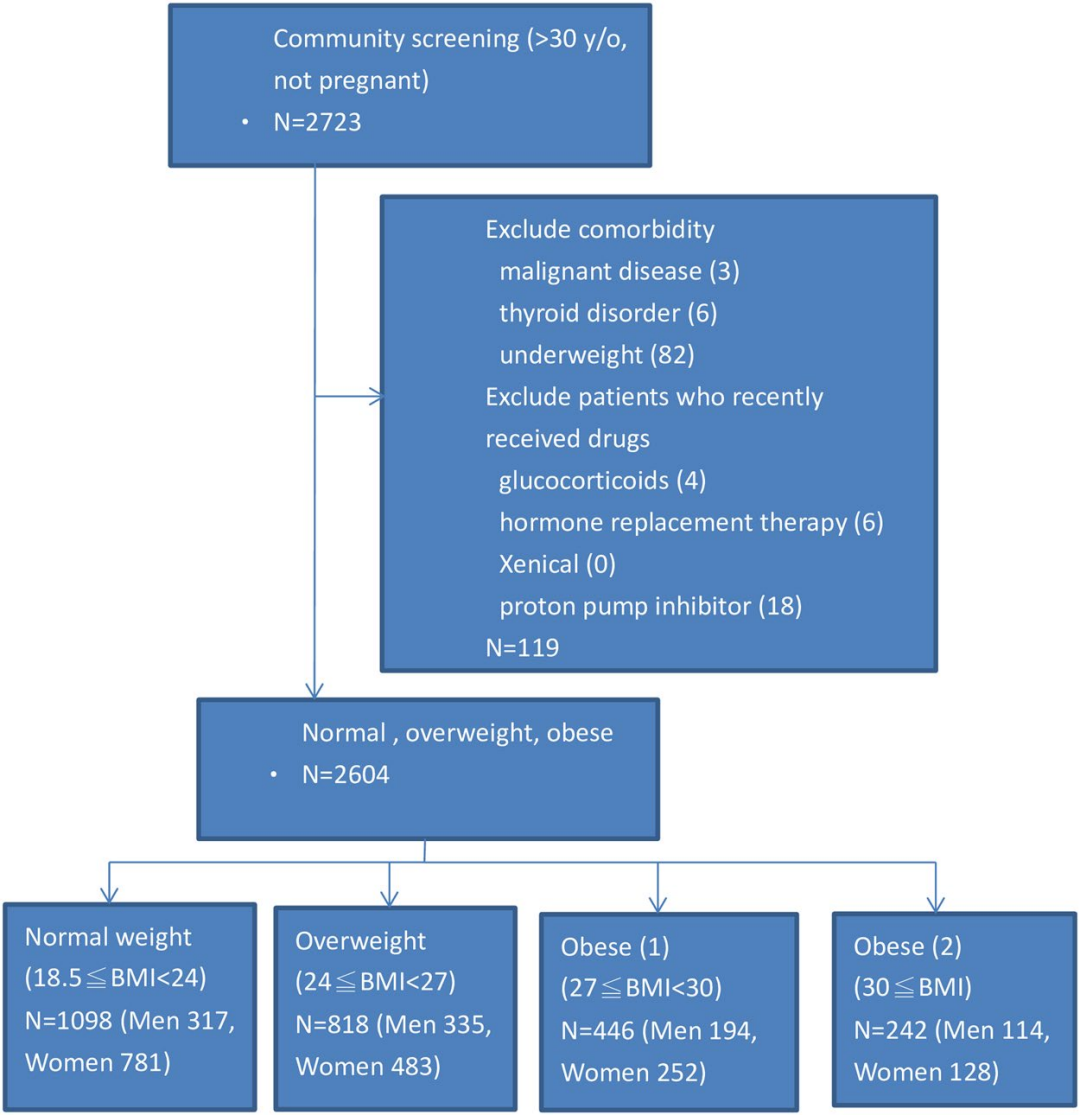
Flow diagram.

**Figure 2:**
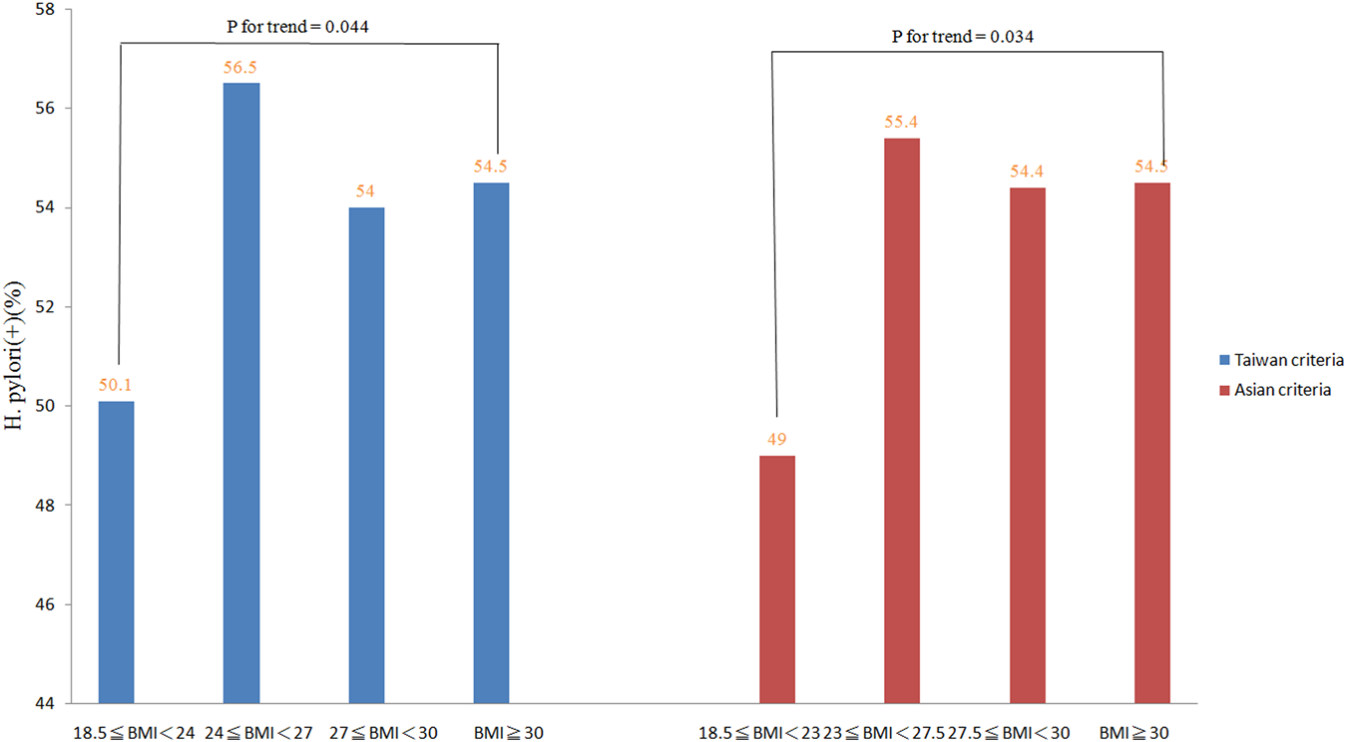
The prevalence of current *H*. *pylori* infection in normal, overweight and obese subjects according to Taiwan and Asian BMI criteria. The blue bars (Taiwan criteria) indicate the prevalence of *H*. *pylori* infection in normal (18.5 ≤ BMI < 24), overweight (24 ≤ BMI < 27), obese 1 (27 ≤ BMI < 30) and obese 2 (BMI ≥ 30, WHO criteria) subjects (*P* for trend = 0.034). The red bars (Asian criteria) indicate the prevalence of *H*. *pylori* infection in normal (18.5 ≤ BMI < 23), overweight (23 ≤ BMI <27.5), obese (27.5 ≤ BMI < 30), and morbidly obese (BMI ≥ 30) subjects (*P* for trend = 0.044).

**Figure 3:**
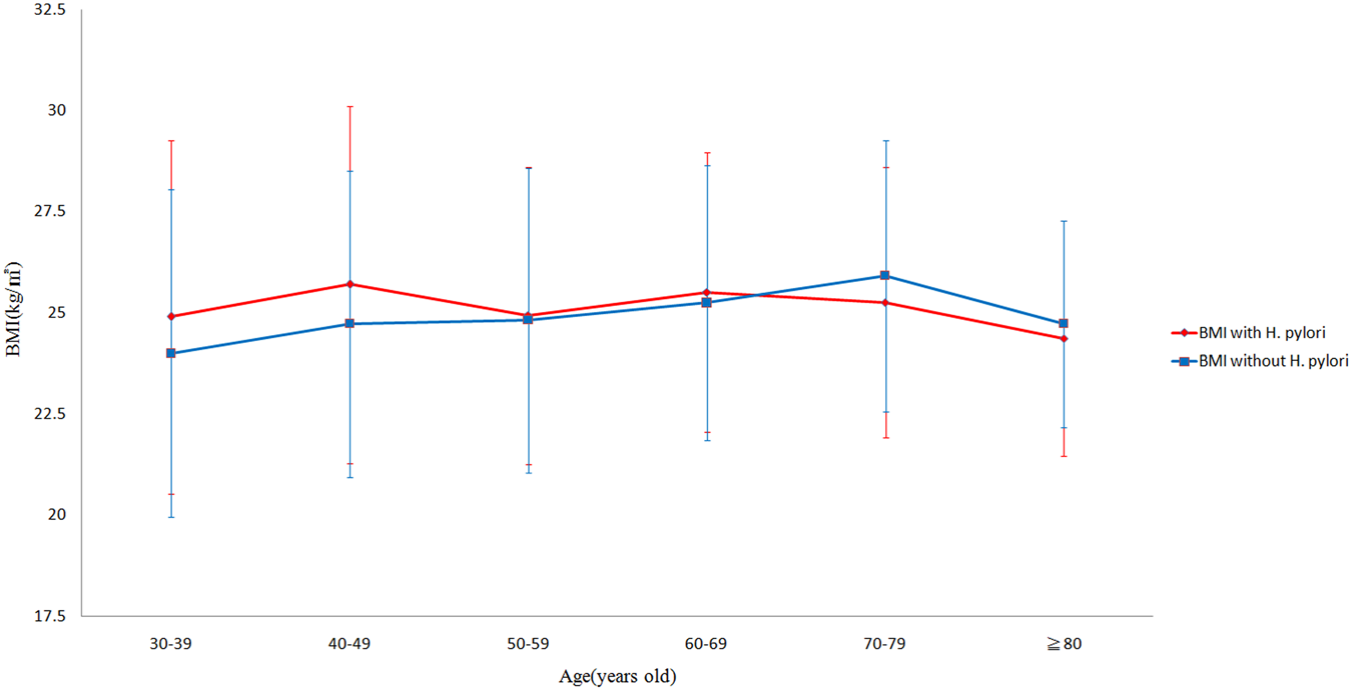
The mean BMI values of the subjects with and without *H*. *pylori* infection for every age period.

A total of 2723 subjects were enrolled. Eighty-two subjects were excluded because their BMIs were less than 18.5 kg/m^2^ (underweight or thin). Thirty-seven subjects were excluded because they had underling diseases or took drugs that would interfere with the *H*. *pylori* test or the accuracy of the BMI calculation. Eighteen subjects had taken proton pump inhibitors, antibiotics or *H*. *pylori* eradication medications within one month, no subjects were taking body weight remodeling drugs (i.e. Xenical), four subjects were taking steroids, six subjects were on hormone therapy, six subjects had underlying diseases or thyroid disorders, and three subjects had underlying malignancies. Ultimately 2604 subjects (1713 women) were included in this study (Fig. 1). These subjects were divided into the following 4 groups by BMI stratification: 1098 subjects in the normal weight group (18.5 ≤ BMI < 24), 818 (31.4%) in the overweight group (24 ≤ BMI < 27), 446 (17.1%) in the obese 1 group (27 ≤ BMI < 30), and 242 (9.3%) in the obese 2 group (BMI ≥ 30). The demographic and characteristic data are listed in Table 1. More than half (57.8%) of the subjects were overweight or obese (BMI ≥ 24). Subjects in the overweight, obese 1 and obese 2 groups were more likely to be male than those in the normal weight group (41%, 43.5%, 47.1%, respectively vs. 28.1%, *P* < 0.001). The prevalence of DM, hyperlipidemia, HTN and metabolic syndrome were higher among the overweight and obese subjects than those of normal weight subjects. The overweight and obese subjects had higher mean HOMA-IR, HS-CRP, leptin, TNF-α, and WBC values and lower mean adiponectin values than those with normal weight. Figure 2 reveals that prevalence of *H*. *pylori* infection in the normal, overweight, obese 1 and obese 2 subjects as classified according to Taiwan’s criteria and the WHO Asian criteria for overweight or obesity. The blue bars (Taiwan criteria) reveal that the prevalence of *H*. *pylori* infection in the normal (18.5 ≤ BMI < 24), overweight (24 ≤ BMI < 27), obese 1 (27 ≤ BMI < 30) and obese 2 (BMI ≥ 30) subjects were 50.1%, 56.5%, 54.0% and 54.5%, respectively (*P* for trend = 0.044). The red bars (Asian criteria) indicated that the prevalence of *H*. *pylori* infection in the normal (18.5 ≤ BMI < 23), overweight (23 ≤ BMI < 27.5), obese 1 (27.5 ≤ BMI < 30) and obese 2 (BMI ≥ 30) subjects were 49.0%, 55.4%, 54.4% and 54.4%, respectively (*P* for trend = 0.034). Figure 3 illustrates that the mean BMI values of the subjects with *H*. *pylori* infection (red line) and without *H*. *pylori* infection (blue line) as stratified by age. In the age periods of 40–49 and 30–39 years, the subjects with *H*. *pylori* infection had higher mean BMI values than those without *H*. *pylori* infection (40–49 years: 25.7 ± 4.4 vs. 24.7 ± 3.8, *P* = 0.025; 30–39 years: 24.9 ± 4.4 vs. 24.0 ± 4.1, *P* = 0.063). Among the subjects aged more than 50 years, there were no significant differences in the mean BMIs between the subjects with and without *H*. *pylori* infection in any age period.

**Table 1:** Demography and Characteristics of Subjects by Body Mass Index Stratification.

Classification	Normal	Overweight	Obese (1)	Obese (2)	*P* value
	18.5 ≤ BMI < 24	24 ≤ BMI < 27	27 ≤ BMI < 30	BMI ≥ 30	
Number (%)	1098(42.2)	818(31.4)	446(17.1)	242(9.3)	
Mean age^†^	56.1 ± 14.0	58.7 ± 13.2	58.9 ± 12.5	55.7 ± 13.6	<0.001
Age stratification					
30–39	180(16.4)	85(10.4)	40(9.0)	36(14.9)	<0.001
40–49	165(15.0)	98(12.0)	60(13.5)	44(18.2)	
50–59	293(26.7)	220(26.9)	118(26.5)	55(22.7)	
60–69	279(25.4)	247(30.2)	139(31.2)	67(27.7)	
70–79	125(11.4)	128(15.6)	70(15.7)	35(14.5)	
≥80	56(5.1)	40(4.9)	19(4.3)	5(2.1)	
Gender					
Male	317(28.9)	335(41.0)	194(43.5)	114(47.1)	<0.001
Female	781(71.1)	483(59.0)	252(56.5)	128(52.9)	<0.001
*H*. *pylori*	550(50.1)	462(56.5)	241(54.0)	132(54.5)	0.044
DM	109(9.9)	134(16.4)	120(26.9)	72(29.8)	<0.001
Waist^†^	74.5 ± 6.4	83.1 ± 6.3	90.0 ± 6.1	97.8 ± 8.2	<0.001
Systolic blood pressure^†^	126.3 ± 19.3	133.0 ± 18.0	137.1 ± 18.3	141.6 ± 16.0	<0.001
Diastolic blood pressure^†^	75.8 ± 11.6	78.9 ± 11.1	81.5 ± 11.4	84.9 ± 10.9	<0.001
FBG^†^	99.2 ± 25.9	103.4 ± 24.2	110.4 ± 33.2	115.9 ± 45.0	<0.001
TG^†^	100.9 ± 102.2	135.6 ± 103.7	150.3 ± 112.8	153.1 ± 93.1	<0.001
HDL^†^	61.1 ± 15.2	54.0 ± 13.7	51.1 ± 12.1	48.0 ± 11.4	<0.001
Metabolic syndrome	100(9.1)	243(29.7)	239(53.6)	170(70.2)	<0.001
Dyslipidemia	905(82.6)	735(90.0)	398(89.2)	219(90.5)	<0.001
HOMA-IR value^†^	1.5 ± 1.8	2.2 ± 2.0	3.1 ± 2.7	4.5 ± 5.6	<0.001
HS-CRP^†^	2.0 ± 5.8	2.4 ± 6.0	2.6 ± 5.2	3.6 ± 5.5	0.002
Adiponectin^†^	9.5 ± 5.3	7.7 ± 4.8	6.2 ± 3.7	6.6 ± 4.8	<0.001
Leptin^†^	8.6 ± 5.6	11.4 ± 7.0	13.4 ± 7.7	16.8 ± 8.8	<0.001
TNF-α^†^	7.4 ± 4.1	7.5 ± 4.2	8.9 ± 11.3	8.5 ± 6.7	0.008
WBC^†^	5.7 ± 1.8	6.2 ± 1.7	6.4 ± 1.7	6.7 ± 1.8	<0.001
SF-36					
PCS^†^	52.7 ± 7.9	52.1 ± 8.0	50.8 ± 8.7	49.5 ± 9.1	<0.001
MCS^†^	49.6 ± 9.5	50.1 ± 10.0	51.4 ± 9.7	52.2 ± 9.5	<0.001
Marriage status					<0.001
Unmarried	102(9.3)	44(5.4)	17(3.8)	26(10.7)	
Married	810(73.8)	653(79.8)	356(79.8)	188(77.7)	
Divorce or Widowed	156(14.2)	102(12.5)	63(14.1)	26(10.7)	
Missing	30(2.7)	19(2.3)	10(2.2)	2(0.8)	
Alcohol	39(3.6)	43(5.3)	19(4.3)	10(4.1)	0.341
Cigarette smoking					0.007
Never	836(76.1)	585(71.5)	308(69.1)	167(69.0)	
Former smoker	104(9.5)	106(13.0)	71(15.9)	37(15.3)	
Current smoker	158(14.4)	127(15.5)	67(15.0)	38(15.7)	

BMI = body mass index, DM = diabetic mellitus, HOMA-IR = homeostasis model assessment of insulin resistance, HS-CRP = high-sensitivity C reactive protein, TNF-α = Tumor necrosis factor alpha, WBC = white blood cell, SF-36 = short form 36, PCS = physical component summary, MCS = mental component summary,

^†^data presented as mean ± standard deviation.

Table 2 reveals the correlations of the category factors, such as demographic variables, lifestyle variables, underlying disease variables, laboratory variables, and physical/psychological score variables, with BMI level and *H*. *pylori infection*. BMI level was positively correlated with demographic factors (age, male and marriage), underlying diseases (DM, hypertension and dyslipidemia), the mental component summary (MCS) score from the SF-36, and laboratory factors (leptin, TNF-α, HS-CRP, HOMA-IR and WBC count). BMI was negatively correlated with the physical component summary (PCS) from the SF-36 and adiponectin value. *H*. *pylori* infection was positively correlated with age, DM, the MCS score from the SF-36 and WBC. *H*. *pylori* infection was negatively correlated with marriage status. The statistically significance factors in the correlation analyses for both BMI level and *H*. *pylori* infection were entered into logistical regression analyses as potential confounding factors. To evaluate the association between *H*. *pylori* infection and the BMI stratification, we estimated the odds ratios for being overweight or obese according to *H*. *pylori* infection status using logistic regression analyses with adjustments for confounding factors and using the control subjects of normal weight (Table 3).Compared with the subjects of normal weight, *H*. *pylori* infection increased the risk of being overweight (OR = 1.226, 95% confidence interval = 1.015–1.480, *P* = 0.034) with adjustments for demographic factors (age, gender and marriage status). A subgroup analysis was performed according to the age of subjects (less or not less than 50 years). Among subjects aged less than 50 years, the adjusted OR for being obese 2 (BMI ≥ 30) was 1.836 (95% CI = 1.079–3.125, *P* = 0.025) for the subjects with *H*. *pylori* infection compared with the subjects without this infection with adjustments for confounding factors.

**Table 2:** Correlation between multivariable, BMI or *H*. *pylori* infection.

Variable^†^	BMI level	*H*. *pylori*
**Demographic variables**		
Age	0.058^*^	0.165^*^
Gender	0.145^*^	0.010
Marriage status	0.103^*^	0.099^*^
**Underlying disease**		
DM	0.195^*^	0.041^*^
Hypertension	0.251^*^	0.033
Dyslipidemia	0.106^*^	0.034
**Physical/mental score**		
SF-36		
PCS	−0.115^*^	−0.017
MCS	0.099^*^	0.064^*^
**Laboratory factors**		
Adiponectin	−0.282^*^	0.002
Leptin	0.322^*^	0.006
TNF-α	0.077^*^	0.047
HS-CRP	0.271^*^	−0.001
HOMA-IR	0.505^*^	0.006
WBC	0.212^*^	0.048^*^

Phi coefficient analysis for category data (*H*. *pylori*, gender, DM, HTN, dyslipidemia) and Spearman’s coefficient rho for rank correlation (BMI level) and Pearson’s correlation coefficient for continuous data (age, PCS, MCS, HS-CRP, TNF-α, adiponectin, leptin, HOMA-IR, WBC). ^*^P < 0.05.

**Table 3:** The Odds ratio of being overweight or obese in subjects with *H*. *pylori* infection than normal control.

Adjust Confounding factors	Adjusted OR (95% CI) of being overweight or obese. Normal control: normal weight					
**All subjects**	overweight vs. normal	*P*-value	Obese (1) vs. normal	*P*-value	Obese (2) vs. normal	*P*-value
Demographic variables	1.226(1.015–1.480)	0.034	1.113(0.886–1.398)	0.357	1.212(0.909–1.614)	0.190
+Underlying disease	1.228(1.016–1.483)	0.033	1.111(0.883–1.400)	0.369	1.209(0.904–1.616)	0.201
+Physical and psychological	1.227(1.015–1.482)	0.034	1.100(0.873–1.386)	0.418	1.185(0.886–1.587)	0.253
+Laboratory variables	1.189(0.983–1.438)	0.075	1.045(0.827–1.319)	0.713	1.133(0.845–1.521)	0.404
**Subjects aged** <**50 years**						
Demographic variables	1.396(0.948–2.056)	0.091	1.085(0.670–1.758)	0.739	1.836(1.079–3.125)	0.025
+Underlying disease	1.404(0.953–2.068)	0.086	1.056(0.649–1.719)	0.826	1.799(1.051–3.080)	0.032
+Physical and psychological	1.385(0.940–2.042)	0.857	1.046(0.643–1.702)	0.857	1.792(1.044–3.076)	0.034
+Laboratory variables	1.380(0.933–2.040)	0.107	1.017(0.619–1.671)	0.946	1.759(1.019–3.039)	0.043
**Subjects aged ≥50 years**						
Demographic variables	1.143(0.919–1.421)	0.229	1.061(0.816–1.378)	0.659	0.999(0.708–1.409)	0.994
+Underlying disease	1.144(0.919–1.423)	0.229	1.061(0.814–1.384)	0.660	1.000(0.705–1.418)	0.999
+Physical and psychological	1.148(0.923–1.429)	0.215	1.050(0.805–1.370)	0.719	0.983(0.692–1.395)	0.921
+Laboratory variables	1.098(0.880–1.369)	0.409	0.987(0.755–1.291)	0.924	0.920(0.647–1.310)	0.645

The confounding factors were adjusted by a stepwise method: first demographic variable, second add underlying disease on demographic variable, third add physical and psychological score variable on demographic plus underlying diseases, finally including all confounding factors.

OR = odds ratio, CI = 95% confidence interval.

Demographic variables: age, sex, marriage.

Underlying disease variables: DM.

Physical and psychological score variables: MCS.

Laboratory variables: WBC.

Control group: normal weight BMI 18.5–24 kg/m^2^.

## Discussion

In the current study, more than half of the subjects were overweight or obese among those aged 50 years or older. The prevalence of overweight or obesity (BMI ≥ 24) was 56.2%, and the mean age was 55.2 years old in this group. As in a previous report, the current study found that most of the overweight or obese people were male, and they had higher prevalence of insulin resistance or diabetic mellitus, dyslipidemia and metabolic syndrome^5,6^.

The associations between *H*. *pylori* infection and overweight/obesity are still under debate (Table 4). The reasons for inconclusive results are multifactorial and include different subject sources (e.g., young, middle-aged or elderly), *H*. *pylori* detection methods (e.g., serum antibody, urea breath test or histology) and BMI criteria for the normal, overweight and obese categories. The majority of the studies did not exclude the underweight (thin) with BMIs < 18.5 from the normal control group.

**Table 4:** Recent studies of the association between *H*. *pylor*i infection and overweight/obese.

Year	Author, nation, subjects source	Number Mean age (year)	*H*. *pylori* detected method	BMI (kg/m^2^) criteria	*H*. *pylori* and obese association OR (95%CI)	Refs
2000	Rosenstock, Danmark, Community	2913 44.6	Serum IgG Ab	Upper quartile ≥26.8	Positive 1.6 (1.1–2.4)	^49^
2009	Arslan, Turkey, Hospital	214 24.3	Serum IgG Ab	Obese ≥30	Positive 2.11 (1.49–3.00)	^33^
2007	Kopacova, Czech, Community	2436 40.6	UBT	Overweight ≥25 Obese ≥30	Positive overweight 1.31 (1.05–1.64) obese 1.25 (0.99–1.57)	^36^
2008	Thjodleifsson, Sweden, Community	985 42	Serum IgG Ab	Overweight >25	Positive 1.86 (1.34–2.60)	^37^
2014	Yang, Taiwan, Hospital	324 67.6	Histology	Obese ≥27	Positive in elderly, 1.89 (1.04–3.45)	^38^
2015	Zhang, China, Hospital	2050 52.2	UBT	Overweight ≥23 Obese ≥27.5^†^	Positive Overweight 1.25(1.00–1.53) Obese 1.28(1.00–1.61)	^34^
2001	Kawano, Japan, Hospital	155 44.2	Serum IgG Ab	BMI value	No association	^39^
2002	Kyriazanos, Greece, Hospital	224 22.8	Serum IgG Ab	BMI ≥25	No association	^29^
2003	Archimandritis, Greece, Hospital	200 48	Serum IgG Ab	Overweight ≥24 Obese ≥27	No association	^40^
2005	Cho, USA, Community	7003 45.2	Serum IgG Ab	Overweight ≥25	No association	^32^
2005	Ioannou, USA, Community	6724 46.7	Serum IgG Ab	Obese ≥30	No association	^31^
2005	Wu, Taiwan, Hospital	1097 31.9	Serum IgG Ab	Morbid obese ≥35^‡^	Inverse relationship	^13^
2007	Mendez-Sanchez Mexico, Hospital	283 46.4	Histology	BMI value	No association	^41^

^†^Use China BMI criteria for obese (≥28) OR = 1.14 (0.89–1.47) (P > 0.05) ^‡^morbid obesity (BMI ≥ 35) vs. normal weight (BMI < 20).

An analysis of an age- and sex-stratified cross section of the Danish population (n = 2913) found that subjects in the upper fourth of the BMI distribution were slightly more likely to be *H*. *pylori*-seropositive (OR adjusted for socioeconomic factors 1.6, 95% CI: 1.1–2.4)^36^. Arslan *et al*. also found a higher *H*. *pylori* infection rate among a young obese group of Turks (mean 24.3 years) compared to a control group (25.5 years; 57.2% vs. 27.0%) and a significant association between obesity and serum antibody positivity for *H*. *pylori* (OR = 2.11, 95% CI = 1.49–3.00, *P* < 0.001)^33^. Two studies by Kopácová *et al*.^37^ of 2,436 Czech people (mean 40.6 years) and Thjodleifsson *et al*.^38^ of 985 Swedish subjects (mean 42 years) also found significant associations between *H*. *pylori* infection and obesity. Recently, two studies by Yang *et al*.^39^ and Zhang *et al*.^34^ from Taiwan and China, respectively, also reported a higher prevalence of *H*. *pylori* infection among overweight and obese subjects than among those of normal weight. Zhang *et al*. reported a trend of increasing *H*. *pylori* infection rates among normal, overweight and obese subjects (37.36%, 41.88%, 45.77% respectively; *P* for trend = 0.006)^34^.

However, other cross-sectional studies have found no association between *H*. *pylori* colonization and the risk of obesity^29,31,32,40–43^. Kawano *et al*.^40^, Kyriazanos *et al*.^29^, and Archimandritis *et al*.^41^ reported that *H*. *pylori* infection is not related to BMI in Japanese and Greek subjects. Later USA studies by Ioannou *et al*.^31^ and Cho *et al*.^32^ reported no relationship between *H*. *pylori* and overweight/obesity.

The *P* value (0.044) for the difference in the incidence of *H*. *pylori* infection was *P* value for the trend of the 4 study groups, including normal, overweight, obese 1 and obese 2 by Taiwan’s criteria. Similarly, the *P* value was 0.034 by Asian’s criteria. The reason for the marginal difference of *H*. *pylori* infection among these 4 groups was the close *H*. *pylori* infection rates between the groups of overweight (56.5%), obese 1 (54%) and obese 2 (54.5%). When we compare the *H*. *pylori* infection rate between normal group (50.1%) and overweight group (56.5%) separately, the difference in the incidence of *H*. *pylori* infection is very significant (*P* < 0.01). Although the *H*. *pylori* rates in obese1 (54.0%) and obese 2 (54.5%) were higher than the rate in normal group (50.1%), the difference was not significant by Chi-square statistic (*P* > 0.05). The reason may be due to relatively small number in the obese 1 and obese 2 (type 2 error). The other explanation is age distribution. If we classified the age with 10 years interval, we find the most different mean BMI value between subjects with or without *H*. *pylori* infection was in age interval 40–50 years old. The mean BMI of subjects aged within 40–50 years and *H*. *pylori* infection was higher than those without this infection (25.7 ± 4.4 vs. 24.7 ± 3.8, *P* = 0.025). If we just compare the *H*. *pylori* infection rates without age classification between these 4 groups, the incidences of *H*. *pylori* among these 4 groups were only marginal significance. If we divided the subjects by aged less than or more than 50 years old, the difference of *H*. *pylori* infection rate among these 4 groups was more significant in subjects aged less than 50 years. In the current study, we found an association between *H*. *pylori* infection and overweight after adjusting for certain confounding factors including age and sex. Moreover, when analyzing subjects aged less than 50 years, *H*. *pylori* infection was associated with obese2 (BMI ≥ 30 kg/m^2^).This is consistent with our previous finding that *H*. *pylori* infection increased insulin resistance and metabolic syndrome in residents younger than 50 years old^12^. Present study further disclosed *H*. *pylori* infection increasing the risk of being obese2 (BMI ≥ 30) than control group in the subjects aged less than 50 years. Hence, the influence of *H*. *pylori* infection on BMI maybe more prominent in subjects aged less than 50 years, when *H*. *pylori* infection is active and showing more influences on insulin resistance^11,12,44^.

One Taiwanese study from Wu *et al*. found that the *H*. *pylori* frequency is lower among the morbidly obese than normal control subjects (43.7% in 414 subjects with BMIs ≥ 35 vs. 60.0% in 683 subjects with BMIs < 25). These authors found an inverse relationship between *H*. *pylori* and BMI and volunteered the hypothesis that *H*. *pylori* could prevent progression to obesity^15^. Other studies are conflicting and have reported weight gain (increased BMI) after successful *H*. *pylori* eradication^19–21,42^. In the current study, a subgroup analysis of the 42 obese subjects (mean age 52.1 years) with BMIs ≥ 35 kg/m^2^ was also performed, and the *H*. *pylori* infection rate was 59.5%. This infection rate was similar to the rates in the overweight (56.5%) and obese (27 ≤ BMI < 35, 53.90%) subjects but higher than the rate in the normal weight subjects (50.1%). The reasons for the different findings regarding the *H*. *pylori* infection rates among morbid obesity subjects (BMI ≥ 35) between Wu’s study and the current study are multifactorial and include the subject source (hospitalized patients vs. community people), the *H*. *pylori* detecting method (serum antibody test vs. UBT) and different BMI cutoff points for normal controls (BMI ≤ 25 vs. 18.5 ≤ BMI < 24).

The additional information provided by this study is that the effect of *H*. *pylori* on obesity may be different between young subjects (aged less than 50) and older subjects. Because most subjects get *H*. *pylori* infection in young age, the inflammatory responses for *H*. *pylori* infection may be more predominant in this period^9,10,35^. However, old subjects have more comorbidity, which results into more inflammatory responses in the whole body, will attenuate or dilute the effect of *H*. *pylori* infection on obesity, insulin resistance or metabolic syndrome.

There are some limitations in the current study. First, this was a cross-sectional study based on community health screening data, so selection bias cannot be excluded. Second, esophagogastroduodenoscopy was not performed for subjects with *H*. *pylori* infection. Some people with *H*. *pylori* infection experienced atrophic gastritis, dyspepsia and weight loss, but other people with *H*. *pylori* infection did not experience appetite loss or weight change. The causality of *H*. *pylori* infection-induced weight change could not be determined in this study. Third, some results, such as daily exercise time or medicine use, were from questionnaires but not from real tests or medical records. Under or overestimated data would be collected due to unsure memory of subjects. Four, the result of current study could not be generalized beyond our study population and area, because the life pattern or food of our subjects may be different from the residents of other areas.

In conclusion, subjects with *H*. *pylori* infection and age less than 50 years old may increase a risk of being obesity than those without this infection.

## Methods

This study originated from a community-based survey for metabolic syndrome and *H*. *pylori* infection. It was performed in the northeastern region of Taiwan, which included the Wanli, Ruifang and Anle districts, from March 2014 to November 2016. The inclusion criteria were an age ≥ 30 years and the absence of pregnancy. The exclusion criteria were conditions that would interfere with *H*. *pylori* tests or BMI calculation accuracy. Participants were excluded if they were currently or had recently (within one month) received medicines for *H*. *pylori* eradication, body weight remodeling (i.e. Xenical), rheumatoid arthritis or autoimmune diseases (i.e., steroid or immunosuppressant treatment), thyroid disorder and malignancy. A standardized questionnaire was administered to all participants by a trained team of interviewers. The items in the questionnaire involved comprehensive alcohol consumption (amount and duration), smoking and betel nut chewing status, and physical activity (the SF36 health survey and daily activity time). All participants received a demographic survey, a physical examination, a ^13^C urea breath test (UBT) for detecting *H*. *pylori* infection, and blood tests. The demographic survey assessed the family history and the medical history of systemic diseases, such as diabetes mellitus (DM), hypertension, hyperlipidemia, rheumatoid arthritis, autoimmune diseases and malignancy. A survey of medication history included proton pump inhibitor therapy, *H*. *pylori* eradication, antibiotics received within one month, hormone therapy, and steroid or immunosuppressant treatment. The physical examination included measurements of basic vital signs (body temperature, respiratory rate, heart rate, and blood pressure), body weight, body height, and waist girth (circumference). Waist girth was measured at the midline between the lowest margin of the subcostal rib and the upper margin of the iliac crest.

The study was conformed to the ethical guidelines of the Declaration of Helsinki, and was performed with the approval of the ethical committee of the Keelung Chang Gung Memorial Hospital. The Institutional Review Board of the Chang-Gung Memorial Hospital approved this research (IRB No: 103-3886C). All participants agreed to join the study and signed an informed consent form before enrollment into the study.

### Body mass index (BMI)

The BMI was calculated as the weight (kg) divided by squared height (m), and the result was recorded in kg/m^2^. The cutoff points for BMI were adopted as suggested by the health promotion administration of the Ministry of Health and Welfare in Taiwan and included normal (18.5 ≤ BMI < 24), overweight (24 ≤ BMI < 27), and obese (BMI ≥ 27) categories^5^. According to the principal cutoff points for obesity from the WHO, a further analysis of the obese subjects was performed by dividing the obese subjects into obese 1 (27 ≤ BMI < 30) and obese 2 (BMI ≥ 30) groups. Another BMI classification from the WHO Expert Consultation for Asians was also used for the analysis in which normal weight was defined as 18.5 ≤ BMI < 23 kg/m^2^, overweight was defined as 23 ≤ BMI < 27.5 kg/m^2^ and obesity was defined as BMI ≥ 27.5 kg/m^2 ^^45^.

### Metabolic syndrome (MS)

A race-specific waist circumference threshold was applied to prevent a discrepancy in MS prevalence according to the NCEP ATP III criteria^46^.

### Urea breath test (UBT)

*H*. *pylori* infection was detected with the Proto Pylori kit (Isodiagnostika, Canada), which contains 75 mg of ^13^C-urea and additives. The results are expressed as delta over baseline (DOB) based on the comparison of two breath samples that were obtained within a 30-minute interval and analyzed by gas chromatography/isotope ratio mass spectrometry. A local validation test with a DOB cut-off value of 3.5 yielded a sensitivity of 96% (95% confidence interval [CI]: 93%–99%) and a specificity of 98% (95% CI: 93%–102%) according to the manufacturer’s reference.

### Adiponectin and leptin

Serum adiponectin and leptin levels were examined with two commercial kits (Human Total Adiponectin/Acrp30, BioVendor Research and Diagnostic system, Minneapolis, MN; Human Leptin ELISA, Clinical Range, BioVendor Laboratory Medicine, Karasek, Czech Republic) according to the manufacturers’ instructions.

### Tumor necrosis factor alpha (TNF-α)

A quantitative sandwich enzyme immunoassay technique was used for the TNF-α assay according to the manufacturer’s instructions (Immunite 1000 LKNF1, Siemens Medical Solutions Diagnostics, Llanberis, UK).

### Homeostasis model assessment of insulin resistance (HOMA-IR)

The HOMA-IR score was calculated according to the following formula:

$${\rm{fasting}}\,{\rm{plasma}}\,{\rm{insulin}}\,({\rm{mU}}/{\rm{L}})\times {\rm{fasting}}\,{\rm{plasma}}\,{\rm{glucose}}\,({\rm{mmol}}/{\rm{L}})/{\rm{22.5}}.$$

A higher HOMA-IR score indicates a greater tendency for insulin resistance (i.e., a lower insulin sensitivity)^47^.

### Short form-36 (SF-36)

The Chinese version of the SF-36 questionnaire was applied for the quality of life survey^48,49^. A lower score indicates greater disability, and a higher score indicates less disability. Two aggregate summary measures, i.e., the physical component summary (PCS) and the mental component summary (MCS), were also analyzed.

### Statistical methods

For continuous variables, the values are expressed as the means and the standard deviations (SDs). T-test was applied for comparing the mean values of two samples. One-way ANOVA was used for comparing the mean values of multiple samples. Categorical data were analyzed with the chi-square test or the Fisher exact test as appropriate. All statistical tests were 2-tailed. A *P*-value of < 0.05 was considered to indicate a statistically significant difference. Correlation coefficients, such as the Pearson, phi and Spearman rho correlation coefficients were chosen. The phi coefficient analysis was utilized for binary category data (i.e. *H*. *pylori*), the Spearman rho coefficient was used for rank correlations (i.e. normal weight, overweight, obese 1 and obese 2) and the Pearson correlation coefficient was used for continuous data (i.e. HS-CRP, TNF-α, adiponectin, leptin). The statistical analyses were performed using SPSS (version 16.0, SPSS Inc., Chicago, IL) for Windows.

### Ethical Adherence

All procedures performed in studies involving human participants were in accordance with the ethical standards of the institutional and/or national research committee and with the 1964 Helsinki declaration and its later amendments or comparable ethical standards. The Institutional Review Board of the Chang-Gung Memorial Hospital approved this research (IRB No: 103-3886C). All participants agreed to the study conditions and provided informed consent before the enrollment in this study.
